# Etiology of diarrhea by multiplex polymerase chain reaction among young children in the United Arab Emirates: a case-control study

**DOI:** 10.1186/s12879-020-05693-1

**Published:** 2021-01-06

**Authors:** Ahmed R. Alsuwaidi, Klaithem Al Dhaheri, Sania Al Hamad, Junu George, Judy Ibrahim, Ghassan Ghatasheh, Mohammed Issa, Suleiman Al-Hammadi, Hassib Narchi

**Affiliations:** 1 Department of Pediatrics, College of Medicine and Health Sciences, United Arab Emirates University, P. O. Box 17666, Al Ain, UAE; 2 Institute of Child Health, Al Ain Hospital, Abu Dhabi Health Services Company (SEHA), Al Ain, UAE; 3 Department of Pediatrics, Tawam Hospital, Abu Dhabi Health Services Company (SEHA), Al Ain, UAE; 4 Department of Emergency Medicine, Tawam Hospital, Abu Dhabi Health Services Company (SEHA), Al Ain, UAE

**Keywords:** Rotavirus, Multiplex PCR, Attributable fractions, Cryptosporidium, Diarrhea

## Abstract

**Background:**

Little is known about the etiology of childhood diarrhea in the United Arab Emirates (UAE) especially after the introduction of rotavirus vaccines. This study aimed to identify gastrointestinal pathogens in children with diarrhea (cases) and the carriage rate of these pathogens in asymptomatic children (controls).

**Methods:**

Stool samples were collected from 203 cases and 73 controls who presented to two major hospitals in Al Ain city, UAE. Samples were analyzed with Allplex™ Gastrointestinal Full Panel Assay for common entero-pathogens. The association between diarrhea and the isolated pathogens was calculated in a multivariate logistic regression model. The adjusted attributable fractions (aAFs) were calculated for all pathogens significantly associated with cases.

**Results:**

At least one pathogen was identified in 87 samples (42.8%) from cases and 17 (23.3%) from controls (*P* < 0.001). Rotavirus, norovirus GII and adenovirus were significantly more prevalent in cases. Their aAFs with 95% ci are 0.95 (0.64, 1.00) for rotavirus, 0.86 (0.38, 0.97) for norovirus GII and 0.84 (0.29, 0.96) for adenovirus. None of the 13 bacteria tested for were more commonly found in the cases than in controls. Cryptosporidium spp. were more significantly detected in cases than in controls. Co-infections occurred in 27.9% of the children. Viruses and parasites were significantly more likely to occur together only in the cases.

**Conclusions:**

Multiplex PCR revealed high positivity rates in both cases and controls which demand a cautious interpretation. Rotavirus remains the main childhood diarrhea pathogen in UAE. Effective strategies are needed to better control rotavirus and other causative pathogens.

**Supplementary Information:**

The online version contains supplementary material available at 10.1186/s12879-020-05693-1.

## Introduction

Infectious diarrhea is a main reason for children’s visits to clinics and emergency rooms. Globally, it is the fifth leading cause of death among children younger than 5 years particularly in lower-income countries [1]. While the responsible microorganisms include a variety of viruses, bacteria and parasites, the majority of diarrhea episodes do not necessitate antibacterial or antiparasitic treatment. The risk of transmission within a household, at school or throughout the community varies according to the causative microorganism. Thus, identifying the organism responsible for the diarrhea is important; particularly for microorganisms associated with diarrhea outbreaks such as various strains of diarrheagenic *Escherichia coli, Salmonella* spp*., Vibrio cholerae, Clostridium difficile, noroviruses and adenoviruses* [2]*.*

Although it has been shown that, in the United Arab Emirates (UAE), 87% of parents seek medical care for their children when they develop gastroenteritis with 10% of these cases requiring hospitalization with an average length of stay of 2.6 days, little is known of the microbiology of gastroenteritis in this country [3]. A hospital-based surveillance study conducted prior to rotavirus vaccine introduction showed that rotavirus gastroenteritis accounted for 50.3% of all gastroenteritis hospitalizations and predominantly affected children younger than 2 years [4]. Although rotavirus vaccine became part of the national immunization program in 2013, there is a lack of information on the distribution of other microorganisms involved in childhood diarrhea in the country.

Usual laboratory investigations for gastroenteritis focus on salmonella, shigella and viruses such as rotavirus. Other possible microorganisms go therefore undetected. Traditional diagnostic tests such as culture, immunoassay and microscopic examination are time-consuming, require special laboratory setup and often lack sensitivity. Culturing most viruses that cause diarrhea is difficult. Other techniques for virus identification such as electron microscopy and immunoassay demand special expertise which is often lacking in many clinical diagnostic laboratories [5, 6].

Multiplex polymerase chain reaction (PCR) based testing has been recently added to the list of microbiological diagnostic tools for several infectious diseases. It allows rapid and simultaneous amplification of several targets with good sensitivity and specificity [5, 7]. Although used in some tertiary hospitals in the UAE, this method, has not yet been evaluated at a population level, where co-infections, as well as asymptomatic carriage of pathogens may exist. If the use of multiplex PCR extends throughout the UAE health care system, it might lead to an overestimation of positive results for diarrhea pathogens in children who might just be asymptomatic carriers, potentially resulting in unnecessary or inappropriate therapy [8]. The aim of this study is to understand the etiology of acute childhood gastroenteritis following rotavirus vaccine introduction in the UAE using a multiplex PCR. We therefore commenced a case-control study to examine the prevalence of different enteric pathogens among children younger than 5 years of age with diarrhea and compare the findings of those with diarrhea-free children.

## Methods

### Study setting, design and enrollment

The study was conducted at two major government-run referral hospitals (Al Ain and Tawam hospitals) in Al Ain, a large inland city in the Eastern Region of the Emirate of Abu Dhabi, UAE with a population estimates of 766,936 people [9]. The UAE is a federal union of seven distinct States-Emirates with the Emirate of Abu Dhabi being the largest and the political capital of the country. Health care is provided for all nationals as mandated by the constitution. National healthcare indicators are equivalent to those in high-income countries with a total expenditure on health as a percentage of gross domestic product being 4.0 in 2010 [10].

Between December 2017 and April 2019, after obtaining parental informed consent, stool specimens were collected from children less than 5 years of age who presented to the two hospitals with diarrhea, defined as at least three loose stools within the previous 24 h, defined as “cases”. During the same period, stool specimens were collected from children attending the same two hospitals without diarrheal disease in the last 30 days, defined as “controls”. Only one stool sample was collected from each participating child. The exclusion criteria for cases included current antibiotic treatment, chronic conditions (e.g., immune deficiency or cystic fibrosis), long-term immunosuppressive therapy and severe or life- threatening co-morbidity. The exclusion criteria for controls included acute infection (e.g., respiratory infection) and having a sibling recruited as a case or control. We also obtained data on demographics, clinical characteristics and exposures (e.g., history of sick contact with diarrhea) through a brief survey of parents at time of enrollment.

### Laboratory testing

Following collection, fecal specimens were transported on a daily basis to the principal investigator’s research laboratory at the UAE University and stored at − 80 °C until further analysis. An automated nucleic acid extraction instrument (Microlab Nimbus IVD system; Hamilton, Reno, Nevada, USA) was used for the extraction of both RNA and DNA from the stool samples. A weighed aliquot of stool sample was suspended in 1-ml of ASL buffer (Qiagen, Valencia, CA, USA) and incubated for 10 min at room temperature. After clarification by high-speed centrifugation, the sample was loaded into the Nimbus equipment for nucleic acid extraction. After nucleic acid extraction, PCR reactions were set up and run on the CFX96 Real Time Detection System (Bio-Rad, Hercules, CA, USA). For that purpose, we used a multiplex one-step real-time PCR platform, the Allplex™ Gastrointestinal Full Panel Assay (Seegene, Seoul, South Korea) as per manufactures’ instructions [11, 12]. This assay has four panels that test for six viruses, 13 bacteria and six parasites. The viral panel detects adenovirus, astrovirus, norovirus GI, norovirus GII, rotavirus and sapovirus. The first bacterial panel detects Enteroaggregative *E. coli*, EAEC (aggR), Enteropathogenic *E. coli*, EPEC (eaeA), *Escherichia coli* O157 (*E. coli* O157), Enterotoxigenic *E. coli*, ETEC (lt/st), hypervirulent *Clostridium difficile* and Enterohemorrhagic *E. coli*, Shiga toxin-producing *E. coli*, STEC (stx1/2). The second bacterial panel detects Aeromonas spp., Campylobacter spp., *Clostridium difficile* toxin B, Salmonella spp., Shigella spp./ Enteroinvasive *E. coli* (EIEC), Vibrio spp. and Yersinia enterocolitica. The parasitic panel detects Blastocystis hominis, Cryptosporidium spp., Cyclospora cayetanensis, *Dientamoeba fragilis*, *Entamoeba histolytica* and *Giardia lamblia*. The PCR reaction was performed under the following cycling conditions: 20 min at 50 °C for one cycle; 15 min at 95 °C for one cycle; 10 s at 95 °C, 1 min at 60 °C and 30 s at 72 °C for 45 cycles; 10 s at 95 °C, 44 times. Seegene Viewer Software (Seegene, Seoul, South Korea) was used for detection and data analysis. Samples were classified as pathogen positive at a cycle threshold value of < 40 as per manufacturer’s instructions [12].

### Data analysis

The descriptive results were expressed as number of participants and percentages. Proportions were compared with the Chi squared or Fisher exact test for small values. As the continuous variables did not follow a normal distribution (as confirmed by the Shapiro-Wilk test), they were expressed as median values and interquartile range (IQR, i.e. 25th and 75th percentile) and were compared with the non-parametric Kruskal Wallis test.

The association between diarrhea and the isolated pathogens was calculated in a multivariate penalized maximum likelihood logistic regression model (Firth’s method) to take into account results with zero participants. The model was corrected for age as a confounder, and the results were expressed as adjusted odds ratios (aOR) with 95% confidence intervals (ci) and their respective *P* values. When there were zero participants in both cases and controls for an individual pathogen, the model could not be computed and thus no results could be reported.

As the number of controls was significantly less than the cases, although we used a penalized maximum likelihood logistic regression model to adjust for age and gender between cases and controls, we still decided to validate the obtained results by executing the same analyses in a post-hoc frequency age-matching case-control analysis. The cases and controls were age-matched within a range of 6 months and were analyzed in a conditional logistic regression model. The association between diarrhea and the isolated pathogens was calculated in an exact logistic regression model (Cox and Snell method) to take into account results with zero participants. The results were expressed as adjusted odds ratios (aOR) with 95% ci and their respective *P* value. The results of the unmatched and age-matched analyses were compared.

Adjusting for potential confounders in the above model, we also calculated, for each pathogen significantly associated with diarrhea in the logistic model, the adjusted attributable fraction (aAF), which is the proportional reduction in diarrhea that would occur if exposure to the respective pathogen was eliminated. aAF (with 95% ci) was calculated from the aOR, with the Woolf approximation for small samples, using the equation:

$$ aAF=\frac{aOR-1}{aOR.} $$

Co-infection amongst the three groups of pathogens was analysed in a logistic model. We calculated their respective odds ratio (OR), with 95% ci and also analyzed the existence of any interaction amongst them, i.e. if the presence of parasites, for example, increased the likelihood of infection with bacteria or viruses, and so on. For all calculations, the statistical software package STATA version 15 was used (StataCorp, Texas, USA) and a two-tailed *P* value < 0.05 defined statistical significance.

## Results

### Study population

**Fig. 1:**
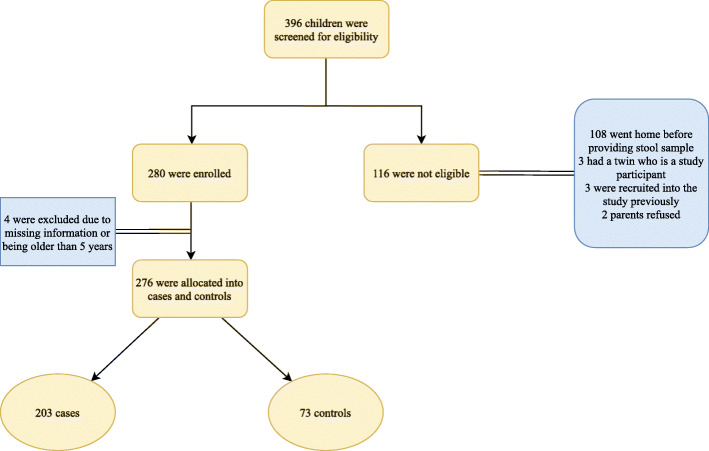
Flowchart of participants recruitment in the study

A total of 276 stool samples were analyzed involving 203 samples from cases and 73 samples from controls (Fig. 1). Cases (median age of 17 months, IQR: 8–23) were significantly older than controls (*P* = 0.01) who had a median age of 11 months (IQR: 2–26). History of sick contact was provided in 30.6% of cases in comparison to approximately 5% in controls (Table 1).

**Table 1:** Demographic and clinical data of 276 enrolled children. Results expressed as numbers (and percentage) unless stated otherwise

	Cases ***n*** = 203	Controls ***n*** = 73	P
Males	101 (49.8)	33 (45.2)	0.50*
Age in months: median (IQR)	17 (8, 23)	11 (2, 26)	0.01^†^
Diarrhea	203 (100)	0 (0)	< 0.001*
*Type*			
Bloody	7 (3.6)	0 (0)	NA
Watery	95 (48.5)	0 (0)	NA
Mucous	54 (27.5)	0 (0)	NA
*Duration (hour)*			
1–96	153 (76.9)	0 (0)	NA
97–120	25 (12.6)	0 (0)	NA
≥ 121	21 (10.5)	0 (0)	NA
*Frequency/24 h*			
1–3	34 (17.1)	0 (0)	NA
4–5	54 (27.1)	0 (0)	NA
≥ 6	111 (55.8)	0 (0)	NA
Vomiting	161 (80.9)	2 (2.8)	< 0.001*
*Duration (hour)*			
1–24	58 (34.5)	0 (0)	NA
25–48	71 (42.2)	0 (0)	NA
≥ 49	39 (23.2)	0 (0)	NA
*Frequency/24 h*			0.2*
1	19 (11.4)	1 (33.3)	
2–4	71 (42.8)	0 (0)	
≥ 5	76 (45.8)	2 (66.7)	
Fever	120 (60.3)	4 (5.5)	< 0.001*
*Duration (hour)*			0.008*
1–24	38 (28.8)	4 (80)	
25–48	59 (44.7)	0 (90)	
≥ 49	35 (26.5)	1 (20)	
*Max recorded fever (*°C)			0.7*
37.1–38.4	71 (55.9)	2 (50)	
38.5–38.9	42 (33.1)	2 (50)	
≥ 39.0	14 (11.0)	0 90)	
Abdominal pain	97 (49.5)	2 (2.7)	< 0.001*
Consumption of meal outside home	19 (9.5)	2 (3.3)	0.11
History of contact	61 (30.6)	3 (4.9)	< 0.001*
Travel abroad in previous 3 months	13 (6.5)	4 (6.5)	0.99

* Chi squared or Fisher exact test; ^†^ Kruskal Wallis test; *IQR* interquartile range; *NA* not applicable

### Pathogen prevalence

A positive PCR result for at least one pathogen was identified more often in 87 samples (42.8%) from cases than in 17 (23.3%) from controls (*P* < 0.001) (Table S1). Overall, the OR of isolating a pathogen, especially a virus (OR = 15.8), were always significantly higher in cases than in controls, in both the unmatched and age-matched multivariate analysis (Table 2). From all the six viruses tested for, only rotavirus, norovirus GII and adenovirus were found significantly more often in the cases than in controls, in both the unmatched and age-matched multivariate analysis (Table 3). However, none of the 13 bacteria tested for were more commonly found in the cases than in controls, in both the unmatched and age-matched multivariate analysis (Table 3). From all the six parasites tested for, only Cryptosporidium was found significantly more often in cases than in controls in the unmatched analysis, but that significance disappeared in the age-matched multivariate analysis (Table 3). Overall, the top three pathogens that were significantly more prevalent in a multivariable logistic regression model include rotavirus, norovirus GII and adenovirus (in a descending order). Their adjusted attributable fractions (aAFs) with 95% ci were 0.95 (0.64, 1.0) for rotavirus, 0.86 (0.38, 0.97) for norovirus GII and 0.84 (0.29, 0.96) for adenovirus (Table S2). Of note, these prevalent pathogens were detected throughout the year (Figure S1). Further, rotavirus and norovirus GII were detected across all studied age groups. However, adenovirus was more prevalent among children younger than 24 months when compared with older children (Table S3).

**Table 2:** Pathogens isolated in the stools of 276 children. Results expressed as number of pathogens (and percentage) unless stated otherwise. (NB. As some stool samples yielded more than one pathogen, the total number of pathogens reported sometimes exceeds the number of participants)

			Univariate Analysis				Multivariate Analysis			
			Unmatched analysis^**a**^		Age-matched analysis^**b**^		Unmatched analysis^**a**^		Age-matched analysis^**b**^	
	Cases ***n*** = 203	Controls ***n*** = 73	aOR (95 ci)	P	aOR (95 ci)	P	aOR (95% ci)	P	aOR (95% ci)	P
Viruses	119 (58.6)	6 (8.2)	15.8 (6.5, 38.1)	< 0.001	14.3 (5.8, 35.4)	< 0.001	18.6 (7.6, 45.6)	< 0.001	16.7 (6.7, 43.0)	< 0.001
Bacteria	84 (41.4)	17 (23.3)	2.3 (1.2, 4.2)	0.009	1.8 (0.9, 3.5)	0.05	2.1 (1.0, 4.2)	0.03	1.8 (0.84, 3.9)	0.1
Parasites	32 (15.8)	2 (2.8)	5.9 (1.3, 25.8)	0.02	10.1 (1.3, 77.7)	0.03	12.15 (2.7, 54.0)	0.01	20.34 (2.5, 166.9)	0.005

^a^ Penalized maximum likelihood logistic regression model, with correction for age; ^b^ Conditional age-matched (6-months blocks) exact logistic regression model; *aOR* adjusted odds ratio; *ci* confidence intervals

**Table 3:** Pathogens isolated in the stool of 276 children. Results expressed as numbers (and percentage) unless stated otherwise

			Unmatched analysis^**a**^			Age-matched analysis^**b**^		
	Cases	Controls	aOR	95% ci	P	aOR	95% ci	P
	***n*** = 203	***n*** = 73						
**Viruses**								
Rotavirus	43 (21.2)	1 (1.4)	19	2.5, 140.0	0.004	21.4	2.8, 161.7	0.003
Norovirus GII	39 (19.2)	2 (2.7)	8.4	1.98, 35.9	0.004	6.9	1.6, 30.1	0.01
Adenovirus	35 (17.2)	2 (2.7)	7.7	1.8, 33.1	0.006	6.1	1.4, 26.9	0.016
Sapovirus	13 (6.4)	2 (2.7)	2.4	0.52, 10.87	0.20	2.4	0.5, 11.9	0.20
Norovirus GI	5 (2.4)	0 (0)	1.4	0.5, 4.3	0.35	1.1	0.2, 12.1	0.66
Astrovirus	4 (1.9)	0 (0)	1.3	0.6, 4.2	0.38	1.1	0.1, 11.8	0.93
**Bacteria**								
Aeromonas spp.	11 (5.4)	1 (1.4)	4.33	0.54, 34.35	0.1	4.71	0.56, 39.7	0.1
EPEC (eaeA)	36 (17.7)	6 (8.2)	2.32	0.93, 5.79	0.07	1.8	0.70, 4.64	0.2
ETEC (lt/st)	7 (3.4)	2 (2.7)	1.2	0.24, 6.0	0.8	1.73	0.32, 9.29	0.5
EAEC (aggR)	21 (10.3)	7 (9.6)	1.04	0.42, 2.57	0.9	0.94	0.37, 2.42	0.9
*Clostridium difficile* toxin B	14 (6.9)	6 (8.2)	0.78	0.28, 2.12	0.6	0.6	0.21, 1.71	0.3
Clostridium difficile hypervirulent	1 (0.5)	1 (1.4)	0.2	0.11, 3.6	0.2	0.65	0.04, 10.4	0.7
Campylobacter spp.	4 (1.9)	0 (0)	1.2	0.6, 4.2	0.4	2.1.	0.19, 14.2	0.5
Salmonella spp.	11 (5.4)	0 (0)	2.1	0.6, 5.0	0.1	3.0	0.46, 15.2	0.2
Shigella spp./EIEC	5 (2.4)	0 (0)	1.2	0.5, 4.1	0.8	1.2	0.09, 20.1	0.8
Yersinia enterocolitica	1 (0.5)	0 (0)	0.2	0.1, 3.4	0.1	0.2	0.006, 19.4	1.0
*E coli* O157	3 (1.5)	0 (0)	1.0	0.1, 4.0	0.4	0.8	0.08, 11.3	1.0
STEC (stx1/2)	2 (0.9)	0 (0)	0.2	0.2, 3.3	0.8	1.2	0.09, 14.7	0.8
**Parasites**								
Cryptosporidium spp.	22 (10.8)	1 (1.4)	7.8	1.02, 59.6	0.04	7.07	0.88, 56.43	0.06
*Dientamoeba fragilis*	3 (1.5)	1 (1.4)	1.01	0.99, 1.04	0.09	1.15	0.10, 12.8	0.9
*Giardia lamblia*	4 (1.9)	0 (0)	1.19	0.2, 4.1	0.8	1.26	0.15, 14.8	0.8
Blastocystis hominis	6 (2.9)	0 (0)	1.30	0.2, 4.2	0.9	4.8	0.58, 18.5	0.1

^**a**^ Penalized maximum likelihood logistic regression model, with correction for age; ^**b**^ Conditional age-matched (6-months blocks) exact logistic regression model; *aOR* adjusted odds ratio; *ci* confidence intervals

### Co-infections

Co-infections occurred in 27.9% of the children, with parasites being present with either viruses or bacteria in 23 children (8.3%). Although amongst all participants, there were no interactions among the three groups of pathogens (all *P* values > 0.05) in the logistic regression analysis (Table 4), viruses and parasites were significantly more likely to occur together only in the cases (Table 4). Cases had a significant higher number of pathogens as compared to controls, with ≥3 pathogens detected in 7.5% of cases, compared with 2.7% of controls (Table S1). The heatmap (Figure S2) shows that EPEC and norovirus GII infection were the most frequent co-infections occurring in 14 children followed by co-infection with EPEC and EAEC in nine children. Of note, EPEC was identified in (17.7%) of cases and (8.2%) of controls (*P* = 0.07).

**Table 4:** Univariate association between the three groups of pathogens isolated in the stools of 276 children

	Cases (***n*** = 203)			Controls (***n*** = 73)		
	aOR*	95% ci	P	aOR*	95% ci	P
Bacterial and parasitic infection	0.96	0.44, 2.07	0.92	0.51	0.05, 5.0	0.56
Viral and parasitic infection	0.21	0.09, 0.49	< 0.001	13.2	0.71, 244.0	0.08
Bacterial and viral infection	1.37	0.77, 2.43	0.27	0.63	0.07, 5.86	0.69

## Discussion

By applying a molecular diagnostic test (i.e., multiplex PCR), this study reveals that in the UAE, rotavirus remains the main pathogen detected in children younger than 5 years of age who present with diarrhea despite the introduction of rotavirus vaccine into the country several years earlier. These results are in agreement with findings from the Global Rotavirus Surveillance Network that included 16 countries in the Americas and Africa regions [13]. In contrast, the introduction of rotavirus vaccines has contributed to ranking norovirus as the leading cause of acute gastroenteritis in U.S. children [14]. As of April 2020, 107 countries have introduced rotavirus vaccines with variable vaccine coverage between high- and low-income countries [15]. Although high-income countries, on average, have lower rotavirus vaccine coverage in comparison to low and lower-middle income countries, the UAE has rotavirus vaccine coverage estimates consistently above 90% for first and last vaccine doses over the past 5 years [16, 17]. Nevertheless, the department of health in Abu Dhabi, UAE was notified of 1144 cases of rotavirus infections in the year 2018 in comparison to only 844 cases in 2017 [18]. Although this increase could reflect improved reporting rather than actual increase in cases, prospective surveillance for rotavirus including evaluation of genotypes distribution remains essential. Moreover, real-world effectiveness of rotavirus vaccines varies according to the setting. Formal evaluation and assessment of the true rotavirus vaccines effectiveness in the UAE population is currently lacking and highly needed.

Norovirus GII was the second prevalent pathogen among the studied children with aAF of 0.86. While the role of norovirus in childhood gastroenteritis is well established in many parts of the world, limited data exist about the contribution of norovirus to the burden of diarrheal disease in the UAE and neighboring countries. A systematic review of studies from 15 out of the 24 countries of the Middle East and North Africa region revealed norovirus infection rates between 0.82 and 36.84% with GII.4 being the most predominant genotype detected [19]. Given recent advances of candidate norovirus vaccines, there is a need for a comprehensive evaluation of local and regional norovirus disease burden including seasonality and strains distribution across the different age groups [20].

Adenoviruses were the third leading cause of diarrhea in our cohort with aAF of 0.84. The clinical course of adenovirus gastroenteritis is often mild and indistinguishable from other viral gastroenteritis. Of note, the Allplex assay is only able to detect enteric adenoviruses belonging to species F (types 40 and 41). Other diarrhea-causing adenovirus species such as A, C and D will be missed when using the Allplex assay [21–24]. This is particularly relevant in the immunocompromised pediatric patients where monitoring adenovirus DNA levels in stools has been suggested to evaluate the benefit of anti-adenovirus pre-emptive therapy [25].

Although none of the 13 bacteria tested for using the Allplex assay were more significantly prevalent in the cases than in controls, diarrheagenic *E. coli* including EPEC, ETEC and EAEC were frequently detected in our studied children. EPEC and EAEC are of debatable diarrheal causation. EPEC is subdivided into typical EPEC (tEPEC) and atypical EPEC (aEPEC) strains based on the presence of EPEC adherence factor plasmid [26]. Recent cumulative data showed that aEPEC are more common than tEPEC. However, given the presence of several virulence factors and association with serious disease, tEPEC is still considered a true pathogen. Nevertheless, it remains unclear if certain aEPEC serotypes are linked with human disease [27]. Further, EAEC has been reported in patients with bloody diarrhea and are probably pathogens. However, it remains unclear if antibiotics are warranted [28]. The 2017 Infectious Diseases Society of America (IDSA) Clinical Practice Guidelines for the Diagnosis and Management of Infectious Diarrhea recommend stool testing for selected bacterial pathogens including *Salmonella, Shigella, Campylobacter, Yersinia*, *C. difficile*, and STEC in people with diarrhea accompanied by fever, bloody or mucoid stools, severe abdominal cramping or tenderness, or signs of sepsis. The IDSA guidelines emphasize that results obtained by culture-independent diagnostic testing, including molecular assays such as multiplex PCR tests, should be cautiously interpreted within the clinical context as these assays may not necessarily detect viable organisms [29].

Cryptosporidium spp. were found significantly more often in cases than in controls in the unmatched analysis, but not in the age-matched multivariate analysis. Cryptosporidium spp. are major cause of gastroenteritis with variable presentations ranging from asymptomatic shedding, self-limiting watery non-bloody diarrhea to a prolonged life-threatening disease in the immunocompromised individuals [30]. The unexpected high rate of Cryptosporidium detection in the studied children raises public health concerns related to potential contamination of drinking water or food. In a study conducted in Sharjah, UAE, 19.4% of stool samples collected from asymptomatic expatriates working in food industry and other domestic occupations during the years 2009 to 2011 were found positive for Cryptosporidium species [31]. In 2018, the department of health in Abu Dhabi, UAE, received nine notifications of Cryptosporidium, all involving children younger than 10 years of age and requiring hospitalization. There was no identifiable source of infection apart from few patients reporting travel history prior to developing symptoms. Of note, reporting Cryptosporidium is not mandatory in the UAE and cases are often notified to health authorities as part of foodborne illnesses investigations [18]. Taken together, we recommend making Cryptosporidium a mandatory reportable disease in the UAE. In addition, further studies are needed to better understand the true prevalence of Cryptosporidium and sources of infection in the country.

Co-infections were found in 27.9% of the studied children. In particular, viruses and parasites were significantly more likely to occur together, but only in the cases. This confirms a previously reported case-control study conducted in southwest China showing that co-infections with two enteric pathogens was higher in cases than in controls (20.1% vs. 5.3%, *P* < 0.05), with rotavirus and norovirus GII being the most common co-infection in symptomatic children and seemingly aggravating the severity of diarrhea [32]. One possible explanation for this aggravation includes the potential for synergistic interaction between the two pathogens resulting in enhanced pathogenicity [33].

This study demonstrates that using molecular multiplex assay is a highly sensitive method for detecting gastrointestinal pathogens. As many pathogens were found in both cases and controls, interpretation of the multiplex PCR results requires a cautious approach. One recommendation is to use a stepwise diagnostic algorithm where screening is initially performed for the pathogens with high aAF; namely rotavirus, norovirus GII and adenovirus. If the initial test is negative, testing for other pathogens is recommended [34]. Alternatively, combining PCR results with the traditional culture results might be useful to ascertain the true viability of the detected pathogen.

This study has few limitations. First, the study was performed only on children presenting exclusively to two local hospitals in Al Ain city who may have different sociodemographic and health characteristics when compared to other children in other parts of the country. Therefore, selection bias is possible, and the study implications cannot be generalized beyond the study settings. Second, although rotavirus was the most predominant pathogen, the lack of knowledge of the children’s rotavirus vaccination status makes it difficult to assess the impact of vaccination on them as well as to evaluate the possibility that positive rotavirus isolates in the studied children could be secondary to recent vaccination. Third, as we have not checked if the controls developed gastrointestinal symptoms days or weeks after the sample collection, introduction of bias cannot be ruled out. One solution would be to make a telephone call 1 month later to establish whether controls had developed acute gastroenteritis after stool sampling, and in the affirmative, exclude them from the analysis. Fourth, controls were younger than cases probably due to the ease of collecting stools from younger children wearing diapers. To overcome this potential bias, we used a logistic regression model to adjust for age and validated the obtained results by executing the same analyses in a post-hoc frequency age-matching case-control analysis. Finally, we had a low number of asymptomatic controls, but this was mitigated by the methods described earlier for the analysis.

## Conclusion

The use of a multiplex PCR has resulted in detection of many pathogens in both cases and controls. Careful interpretation of the test result is therefore needed before making treatment decisions. As rotavirus remains the main pathogen detected among children with diarrhea in UAE, there is a need for better vaccine strategies in the country. Comprehensive evaluation of the true prevalence of Cryptosporidium and sources of infection in the country are warranted.

## Supplementary Information

**Additional file 1 Table S1**. Number of pathogens isolated in the stools of 276 children. **Table S2**. Adjusted attributable fractions (aAFs) for pathogens that were significant in a multivariable logistic regression model. **Table S3**. Prevalence of enteric pathogens in of 276 enrolled children (203 cases and 73 controls) by age group. **Figure S1**. Monthly detections of the most prevalent pathogens among 276 enrolled children. **Figure S2**. Number of participants with co-infections with gastrointestinal pathogens in 276 enrolled children.

## Data Availability

The datasets used and/or analyzed during the current study are available from the corresponding author on reasonable request.

## Abbreviations

UAE: United Arab Emirates
PCR: Polymerase chain reaction
EAEC (aggR): Enteroaggregative *E. coli*
EPEC (eaeA): Enteropathogenic *E. coli*
*E. coli* O157: *Escherichia coli* O157
ETEC (lt/st): Enterotoxigenic *E. coli*
EIEC: Enteroinvasive *E. coli*
STEC (stx1/2): Shiga toxin-producing *E. coli*
IQR: Interquartile range
OR: Odds ratio
aOR: Adjusted odds ratios
ci: Confidence intervals
aAF: Adjusted attributable fraction
aEPEC: Atypical Enteropathogenic *E. coli*
tEPEC: Typical Enteropathogenic *E. coli*
IDSA: Infectious Diseases Society of America
