# Supplementary material for: Etiology of diarrhea by multiplex polymerase chain reaction among young children in the United Arab Emirates: a case-control study
Source: BMC Infect Dis. 2021 Jan 6;21:7. doi: 10.1186/s12879-020-05693-1 (PMC7788778; doi:10.1186/s12879-020-05693-1)
Supplement: Supplementary file 1 — Additional file 1 Table S1. Number of pathogens isolated in the stools of 276 children. Table S2. Adjusted attributable fractions (aAFs) for pathogens that were significant in a multivariable logistic regression model. Table S3. Prevalence of enteric pathogens in of 276 enrolled children (203 cases and 73 controls) by age group. Figure S1. Monthly detections of the most prevalent pathogens among 276 enrolled children. Figure S2. Number of participants with co-infections with gastrointestinal pathogens in 276 enrolled children. [file 12879_2020_5693_MOESM1_ESM.docx]

**Supplementary Materials:**

| **Table 1S.** Number of pathogens isolated in the stools of 276 children | | | | |
| --- | --- | --- | --- | --- |
|  | **Number of pathogens** | **Cases**  **n=203** | **Controls n=73** | **P** |
| **Viruses** |  |  |  | <0.001 |
|  | 0 | 84 (41.4) | 67 (91.8) |  |
|  | 1 | 102 (50) | 5 (6.8) |  |
|  | 2 | 15 (7.4) | 1 (1.4) |  |
|  | 3 | 1 (0.5) | 0 (0) |  |
|  | 4 | 1 (0.5) | 0 (0) |  |
|  |  |  |  |  |
| **Bacteria** |  |  |  | 0.07 |
|  | 0 | 119 (58.6) | 56 (76.7) |  |
|  | 1 | 63 (31) | 13 (17.8) |  |
|  | 2 | 13 (6.4) | 3 (4.1) |  |
|  | 3 | 5 (2.4) | 0 (0) |  |
|  | 4 | 3 (1.4) | 1 (1.4) |  |
|  |  |  |  |  |
| **Parasites** |  |  |  | 0.01 |
|  | 0 | 171 (84.2) | 71 (97.2) |  |
|  | 1 | 29 (14.3) | 2 (2.7) |  |
|  | 2 | 3 (1.5) | 0 (0) |  |
|  |  |  |  |  |
| **Total pathogens** |  |  |  | <0.001 |
|  | 0 | 38 (18.7) | 50 (68.5) |  |
|  | 1 | 87 (42.8) | 17 (23.3) |  |
|  | 2 | 47 (23.1) | 4 (5.5) |  |
|  | 3 | 18 (8.9) | 1 (1.4) |  |
|  | 4 | 10 (4.9) | 1 (1.4) |  |
|  | 5 | 3 (1.5) | 0 (0) |  |

| **Table 2S. Adjusted attributable fractions (aAFs) for pathogens that were significant in a multivariable logistic regression model** | | | | | | |
| --- | --- | --- | --- | --- | --- | --- |
|  |  |  | **Age-matched analysis*** | | |  |
|  | **Cases** | **Controls** | **OR** | **95% ci** | **P** | **aAF (95% ci)^§^** |
|  | **n= 203** | **n=73** |  |  |  |  |
| Rotavirus | 43 (21.2) | 1 (1.4) | 21.4 | 2.8, 161.7 | 0.003 | 0.95 (0.64, 1.00) |
| Norovirus GII | 39 (19.2) | 2 (2.7) | 6.9 | 1.6, 30.1 | 0.01 | 0.86 (0.38, 0.97) |
| Adenovirus | 35 (17.2) | 2 (2.7) | 6.1 | 1.4, 26.9 | 0.016 | 0.84 (0.29, 0.96) |
|  |  |  |  |  |  |  |
| *Conditional age-matched (6-months blocks) logistic regression  **^§^** aAF: adjusted attributable fraction with Woolf approximation | | | | | | |

**Table 3S. Prevalence of enteric pathogens in of 276 enrolled children (203 cases and 73 controls) by age group.**

|  | **Age: <12 months**  **n= 107** | | |  | **Age: 12-23 months**  **n=97** | | |  | **Age: 24-59 months**  **n=72** | | |
| --- | --- | --- | --- | --- | --- | --- | --- | --- | --- | --- | --- |
|  | **Cases** | **Controls** | ***P*** * |  | **Cases** | **Controls** | ***P*** * |  | **Cases** | **Controls** | ***P*** * |
|  | **n=69 (%)** | **n=38 (%)** |  |  | **n=84 (%)** | **n=13 (%)** |  |  | **n=50 (%)** | **n=22 (%)** |  |
| **Norovirus GI** | 2 (2.9) | 0 (0) | 0.5 |  | 2 (2.4) | 0 (0) | 1.0 |  | 1 (2.0) | 0 (0) | 1.0 |
| **Norovirus GII** | 11 (15.9) | 1 (2.6) | 0.05 |  | 18 (21.4) | 1 (7.7) | 0.40 |  | 10 (20.0) | 0 (0) | 0.026 |
| **Rotavirus** | 9 (13.0) | 1 (2.6) | 0.09 |  | 22 (26.2) | 0 (0) | 0.036 |  | 12 (24.0) | 0 (0) | 0.013 |
| **Adenovirus** | 14 (20.3) | 1 (2.6) | 0.017 |  | 16 (19.0) | 0 (0) | 0.11 |  | 5 (10) | 1 (4.5) | 0.66 |
| **Astrovirus** | 3 (4.3) | 0 (0) | 0.55 |  | 1 (1.2) | 0 90) | 1.0 |  | 0 (0) | 0 (0) | N/A |
| **Sapovirus** | 5 (7.2) | 0 90) | 0.15 |  | 4 (4.8) | 1 (7.7) | 0.52 |  | 4 (8.0) | 1 (4.5) | 1.0 |
| **Campylobacter spp*.*** | 1 (1.4) | 0 (0) | 1.0 |  | 3 (3.6) | 0 (0) | 1.0 |  | 0 (0) | 0 (0) | N/A |
| **Clostridium difficile toxin B** | 1 (1.4) | 2 (5.2) | 0.28 |  | 8 (9.5) | 3 (23.1) | 0.16 |  | 5 (10.0) | 1 (4.5) | 0.66 |
| **Salmonella spp.** | 3 (4.3) | 0 (0) | 0.55 |  | 6 (7.1) | 0 (0) | 1.0 |  | 2 (4.0) | 0 (0) | 1.0 |
| **EIEC shigella** | 0 (0) | 0 (0) | N/A |  | 2 (2.4) | 0 (0) | 1.0 |  | 3 (6.0) | 0 (0) | 0.54 |
| **Vibrio spp.** | 0 (0) | 0 (0) | N/A |  | 0 (0) | 0 (0) | N/A |  | 0 (0) | 0 (0) | N/A |
| **Yersinia enterocolitica** | 1 (1.4) | 0 (0) | 1.0 |  | 0 (0) | 0 (0) | N/A |  | 0 (0) | 0 (0) | N/A |
| **Aeromonas spp.** | 6 (8.7) | 0 (0) | 0.08 |  | 2 (2.4) | 1 (7.7) | 0.35 |  | 3 (6.0) | 0 (0) | 0.54 |
| **Clostridium difficile hypervirulent** | 0 (0) | 0 (0) | N/A |  | 0 (0) | 0 (0) | N/A |  | 1 (2.0) | 1 (4.5) | 0.52 |
| ***E. Coli* O157** | 0 (0) | 0 (0) | N/A |  | 3 (3.6) | 0 (0) | 1.0 |  | 0 (0) | 0 (0) | N/A |
| **STEC (stx1/2)** | 0 (0) | 0 (0) | N/A |  | 0 (0) | 0 (0) | N/A |  | 2 (4.0) | 0 (0) | 1.0 |
| **EPEC (eaeA)** | 8 (11.6) | 1 (2.6) | 0.15 |  | 19 (22.6) | 2 (15.4) | 0.72 |  | 9 (18.0) | 3 (13.6) | 0.74 |
| ***ETEC (It/st)*** | 3 (4.3) | 1 (2.6) | 1.0 |  | 2 (2.4) | 0 (0) | 1.0 |  | 2 (4.0) | 1 (4.5) | 1.0 |
| ***EAEC (aggR)*** | 9 (13.0) | 2 (5.2) | 0.32 |  | 7 (8.3) | 1 (7.7) | 1.0 |  | 5 (10) | 4 (18.2) | 0.44 |
| **Giardia lamblia** | 1 (1.4) | 0 (0) | 1.0 |  | 2 (2.4) | 0 90) | 1.0 |  | 1 (2.0) | 0 (0) | 1.0 |
| ***Cryptosporidium spp.*** | 2 (2.9) | 1 (2.6) | 1.0 |  | 11 (13.1) | 0 (0) | 0.35 |  | 9 (18.0) | 0 (0) | 0.049 |
| **Blastocystis hominis** | 0 (0) | 0 (0) | N/A |  | 2 (2.4) | 0 (0) | 1.0 |  | 4 (8.0) | 0 (0) | 0.30 |
| **Dientamoeba fragilis** | 0 (0) | 0 (0) | N/A |  | 0 (0) | 0 (0) | N/A |  | 3 (6.0) | 1 (4.5) | 1.0 |
| **Cyclospora cayeanensis** | 0 (0) | 0 (0) | N/A |  | 0 (0) | 0 (0) | N/A |  | 0 (0) | 0 (0) | N/A |
| * P value by Fisher exact test; N/A: not applicable  Enteroaggregative *E. coli*: EAEC (aggR); Enteropathogenic *E. coli*: EPEC (eaeA); *Escherichia coli* O157: *E. coli* O157; Enterotoxigenic *E. coli*: ETEC (lt/st); Enterohemorrhagic *E. coli*, Shiga toxin-producing *E. coli*: STEC (stx1/2) | | | | | | | | | | | |

**Fig 1S.** Monthly detections of the most prevalent pathogens among 276 enrolled children.

**
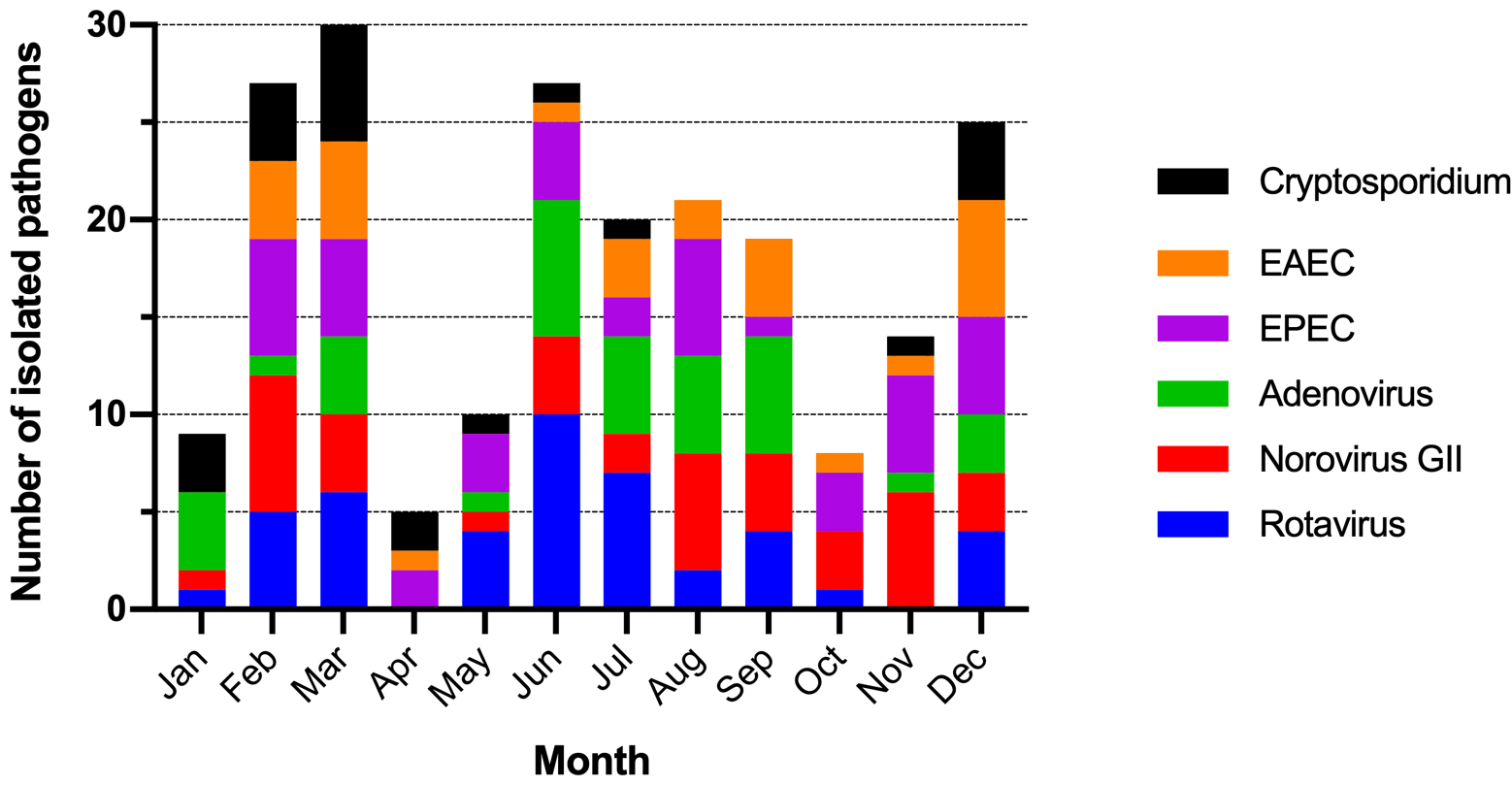
**

**Fig 2S.** Number of participants with co-infections with gastrointestinal pathogens in 276 enrolled children.


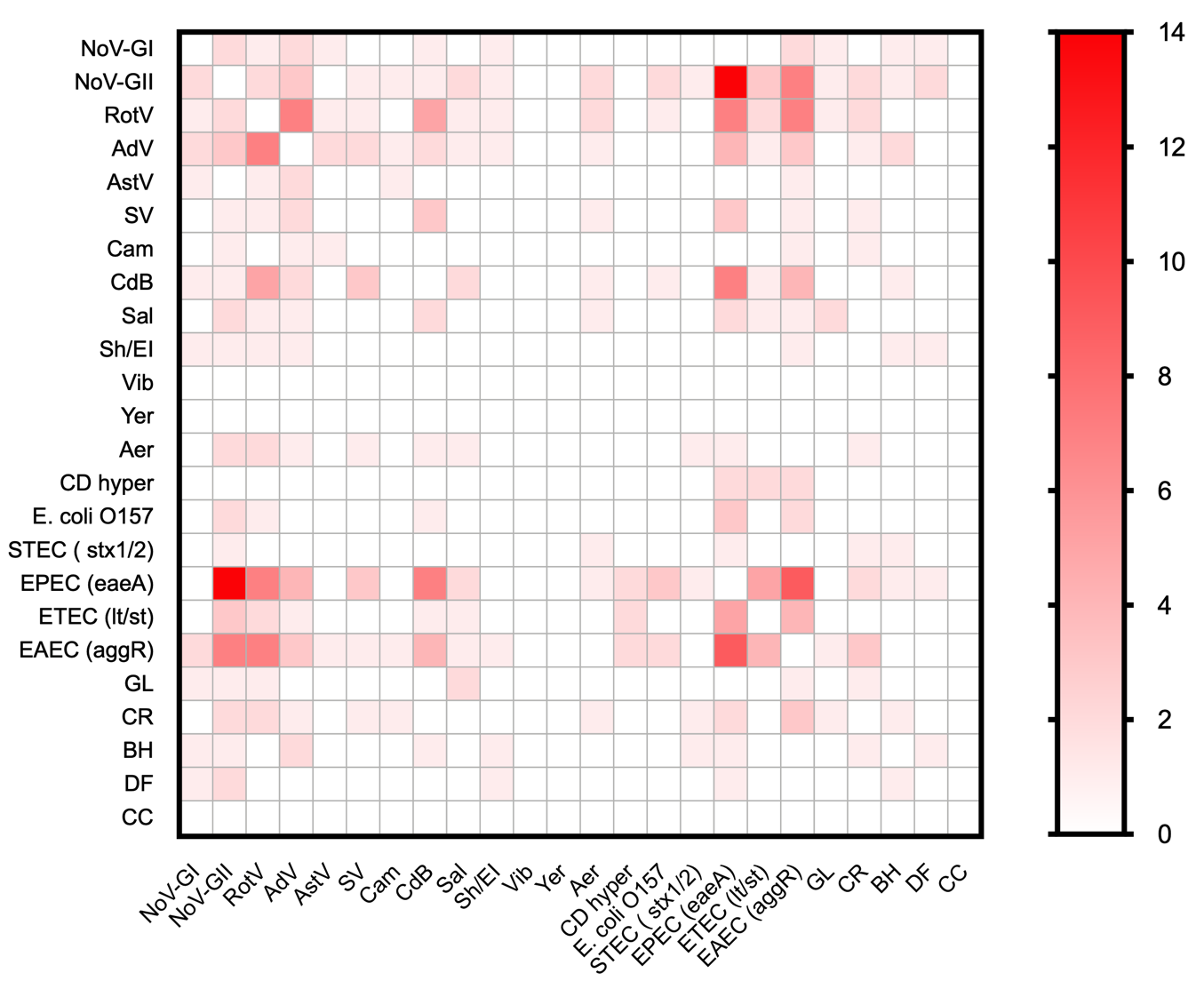


NoV-GI: Norovirus GI; NoV-GII: Norovirus GII; RotV: Rotavirus; AdV: Adenovirus;

AstV: Astrovirus; SV: Sapovirus; Cam: *Campylobacter* spp.; CdB: *Clostridium difficile* toxin B; Sh/EI: *Shigella* spp./Enteroinvasive *Escherichia coli*; Vib: *Vibrio* spp.; Sal: *Salmonella* spp.; Yer: *Yersinia enterocolitica*; Aer: *Aeromonas* spp.; CD hyper: Hypervirulent *Clostridium difficile*; E. coli O157: *Escherichia coli* O157; STEC (stx1/2): Shiga toxin-producing *E. coli*; EPEC (eaeA): Enteropathogenic *E. coli*; ETEC (It/st): Enterotoxigenic *E. coli*; EAEC (aggR): Enteroaggregative *E. coli*; GL: *Giardia lamblia*; CR: *Cryptosporidium* spp.; BH: *Blastocystis hominis*; DF: *Dientamoeba fragilis* ; CC: *Cyclospora cayetanensis*
